# Development of a new computer simulated environment to screen cognition: assessing the feasibility and acceptability of Leaf Café in younger and older adults

**DOI:** 10.1186/s12911-024-02478-3

**Published:** 2024-03-19

**Authors:** Joyce Siette, Jonathan Guion, Kiran Ijaz, Paul Strutt, Meredith Porte, Greg Savage, Deborah Richards

**Affiliations:** 1 The MARCS Institute for Brain, Behaviour and Development, Western Sydney University, Westmead, NSW 2145 Australia; 2 Australian Institute of Health Innovation, Macquarie University, Macquarie Park, NSW 2109 Australia; 3 Affective Interactions Lab, School of Architecture, Design and Planning, University of Sydney, Sydney, NSW Australia; 4 School of Psychology, Western Sydney University, Kingswood, NSW 2747 Australia; 5 School of Computing, Macquarie University, Macquarie Park, NSW 2109 Australia; 6 School of Psychological Sciences, Macquarie University, Macquarie Park, NSW 2109 Australia

**Keywords:** Virtual reality, Dementia, Cognition, Screening, Performance, Technology

## Abstract

**Background:**

Existing traditional cognitive screening tools for dementia have various limitations, including overreliance on tests assessing verbal memory and, to a lesser extent, on some aspects of executive functioning. Comprehensive neuropsychological assessment is sensitive to impairment but time-intensive and expensive. Virtual reality may provide a dynamic and unique understanding of cognitive performance and increase the ecological validity of cognitive assessment. The use of virtual reality in screening for cognitive function in older persons is promising, but evidence for its use remains sparse.

**Objective:**

Our primary aim was to examine the feasibility and acceptability of a newly developed, virtual reality assessment module, ‘Leaf Café’, a computer-based program that assesses cognition in an engaging, efficient, and ecologically relevant way. The secondary aim was to assess the ability of the module to discriminate between performances of younger and older adults.

**Methods:**

A cross-sectional study was carried out in Sydney, Australia, targeting adults aged 18 years and above. Participants completed a traditional cognitive screening tool (Telephone Interview for Cognitive Status-Modified, TICS-M) and Leaf Café, a low-immersive virtual reality module designed to evaluate learning and memory, perceptual-motor function, and executive functioning. The total performance score for each participant, ranging from 0 to 180, was correlated with their cognitive performance assessed by TICS-M, using Pearson’s correlation coefficient. Following module completion, participants were presented with an open and closed-question survey to capture their perceptions, attitudes, and feedback on the module, encompassing practicality, acceptability, and enjoyment. Both descriptive and content analyses were employed to interpret the obtained data.

**Results:**

A sample of 131 participants (mean age 54.9 years, SD = 20.8, range 20–85) took part. The majority were female (71.8%) and born in an English-speaking country (75.8%). The mean amount of time spent in the module was 32.8 min (SD = 13.3) with a mean module score of 107.6 (SD = 38.7). Most participants completed the highest level (5; 80.5%). There was a significant correlation between Leaf Café total scores with TICS-M cognitive scores overall, and for both younger (aged 18–64 years) and older adult (aged 65 + years) groups. No significant difference was found on performance between age groups on TICS-M performance, however, younger adults had significantly better performance on the Leaf Café module than older adults (M = 124.1 vs 95.9; *p* < .001). Participants had similar response proportions regarding user experience with most agreeing that the module was easy to use (84%) and to navigate (85%). Compared with younger adults, older adults had lower rates of agreement on the module’s design (36.8% vs 64.3%; *p* = .020) and support experienced (20.5% vs 53.6%; *p* = .007). Participants highlighted the significance of practicality and the cognitive challenges presented by the module, in terms of memory strain and user interface concerns. Feedback encompassed different opinions on the usefulness of music, with suggestions for improvements centred around clearer instructions, varied game dynamics, and considerations for diverse user needs.

**Conclusions:**

Leaf Café is a feasible and acceptable tool to be used for screening for cognitive impairment in older adults and has real-world assessment value. Further verification on the game’s utility in detecting cognitive impairment is required.

**Supplementary Information:**

The online version contains supplementary material available at 10.1186/s12911-024-02478-3.

## Introduction

Dementia is a global health priority. The World Health Organization reports that dementia currently affects more than 50 million individuals globally, and the number is expected to increase to 82 million by 2030 [1]. However, diagnosing dementia accurately is restricted by the lack of sensitive and reliable screening tools, which can lead to potential misdiagnoses or late diagnosis [2]. Having a valid, reliable, and well-constructed cognitive screener for detecting cognitive decline would contribute to improving opportunities for intervention in addition to decreasing patient and caregiver burden. Furthermore, earlier diagnoses are associated with net positive outcomes for the individual experiencing dementia as they permit the individual and their carers to plan for help, support, and treatments more adequately [3]. Given that new disease-modifying treatments, such as Lemanacab, have recently obtained approval for the treatment of Alzheimer’s disease in the USA [4], this heightens the significance of early disease detection, facilitating timely prescription of medication during the initial stages of disease progression.

Cognitive screening tools can assist with early detection of impaired cognitive function and through repetitive screening, provide quantifiable, predictive data for healthcare professionals to evaluate cognitive performance [5]. Such tools are already widely used in primary care settings, such as the Mini-Mental State Exam (MMSE) [6], Montreal Cognitive Assessment (MoCA) [7], the Ascertain Dementia 8 Questionnaire (AD-8) [8], Mini-cog [9], and clock drawing test [10], which allows healthcare professionals to detect the presence of cognitive impairment and rate of cognitive decline [11]. However, these screening tools have several limitations, including ceiling effects [12] and elevated chance of false positives [13], and are restricted to testing certain functions [14], which can impact on the accuracy of dementia detection.

Recent developments of virtual reality (VR) technologies to measure cognitive function show promise. First introduced in 1989, computers were used in early attempts to develop realistic scenes using sensor equipment to support users with training and learning in real-time environments that were not previously possible to experience conveniently [15]. The emergence of VR technologies became particularly useful within clinical research, supporting surgical simulation and medical training for medical students using virtual patients to avoid potentially adverse outcomes [16].

Exploring the use of virtual environments to support diagnosis and early detection of cognitive impairment in older adults has gained interest. Systematic reviews support VR as a versatile tool for diagnosing, screening, and providing therapeutic interventions for dementia status [17, 18] with studies investigating the use of VR to screen for cognitive impairment in older adults [19, 20]. Reviews highlight that VR could potentially identify and support people with cognitive impairment, however, prior reviews focused on immersive VR applications (e.g., [17]), which are prone to self-reported side-effects through locomotion [21] and studies reviewed had limited sample sizes.

Ongoing investigations into using VR as a cognitive diagnostic tool are at an early stage, with a limited number of studies conducted thus far [22]. These studies often lack comparisons with younger cohorts, which may limit adaptability of these modules for use over the lifespan, and have small sample sizes [23–25]. Despite this, VR technology may provide a highly sensitive screening tool for distinguishing between participants with or without early memory impairments, with a recent accurate classification rate of 87.3% [26]. Although the advantages of VR-based technologies for older adults are promising, perceptions of diagnostic-based VR in terms of usability and acceptability remains largely unexplored.

Older adults tend to indicate openness to innovative digital technologies and a willingness to use new technologies [27, 28], especially if they perceive it as beneficial [29]. Comparable findings have been observed concerning attitudes toward future VR usage, provided the technology is user-friendly and offers a positive experience [30]. However, there is still a high level of technology rejection amongst older adults, with anxiety associated with virtual three-dimensional experiences when using VR equipment contributing to decreased acceptance [31]. Additionally, older adults often report feeling “lightheaded” after prolonged use of VR games [32]. To ensure older adults are comfortable using VR technology, it is important that the VR platform itself is easy to use and navigate for older adults [33].

Our study thus aimed to (i) evaluate the feasibility and acceptability of our newly developed low-immersive virtual reality module (Leaf Café) as a screening tool for cognitive functioning in younger and older adults; and (ii) determine its ability to discriminate between younger and older participants.

## Methods

### Study design

A cross-sectional study comprising eligible adults aged 18 years or older participated in three activities between March 2021 to August 2022: (i) the virtual reality module, (ii) a traditional cognitive screening assessment, and (iii) an online survey assessing the acceptability and feasibility of the virtual reality module.

### Participants and eligibility

Participants were recruited from multiple sources including existing researcher mail lists, social media, convenience and snowball sampling, flyer distribution, and other organizational contacts from the research team. Participants were excluded if they were aged < 18 years old, had a self-reported diagnosis of dementia, aphasia, or severe depression, or did not have internet access or an electronic device to open the module (e.g., a PC or a laptop). Participants were required to understand the procedure for using the Leaf Café module and have the capacity to provide informed consent. Participants who had poor vision (self-reported), inability to follow verbal commands, or inability to comply with the study were also excluded. All participants provided informed consent prior to their participation in the study. This study was approved by the Macquarie University Ethics Committee (reference 52020917422912) and all procedures were performed in accordance with the Declaration of Helsinki.

### General procedure

Participants accessed the cafe module via a provided hyperlink accessed through the internet. Informed consent was obtained through the introductory page before proceeding to the module. Participants then completed the module on their own devices in their own time. Participants were then contacted within two weeks to complete the Telephone Interview for Cognitive Status-Modified (TICS-M). Participants were asked to provide responses on their own, without help from others and to not take notes or look up answers.

After completion of the module, participants were invited to provide feedback via a voluntary online survey about the feasibility and acceptability of their virtual reality experience.

### Measures

#### Cognitive function

Classification of cognitive status was conducted by administering the 23-question TICS-M over the phone. This screener included items regarding orientation (person, place, and time), attention and working memory (counting backwards, serial 7’s), language (object naming, concept formation, simple phrase repetition, and comprehension of instructions), and memory (immediate and delayed recall of a 10-item word list). The maximum score was 39 points and individuals were classified as having possible cognitive impairment with a cut-off score ≤ 21. The TICS-M has previously been validated [34] with strong psychometric properties [35]. Prior studies have shown that the TICS-M has a high sensitivity in the detection of dementia [29, 36] but a low positive predictive value [34]. The TICS-M shows good test–retest reliability and high inter-rater reliability [37], as well as excellent sensitivity (99%) and specificity (86%), and is considered suitable for early screening of cognitive impairment [38].

#### Assessment module

Developed using the Unity game engine, the application places the user in a virtual restaurant modelled to be similar to a café, in the role of a waiter serving customers. The module was developed using Unity’s WebGL build option to allow the application to run in current versions of most major web browsers (e.g., Microsoft Edge, Mozilla Firefox, and Google Chrome).

**Fig. 1:**
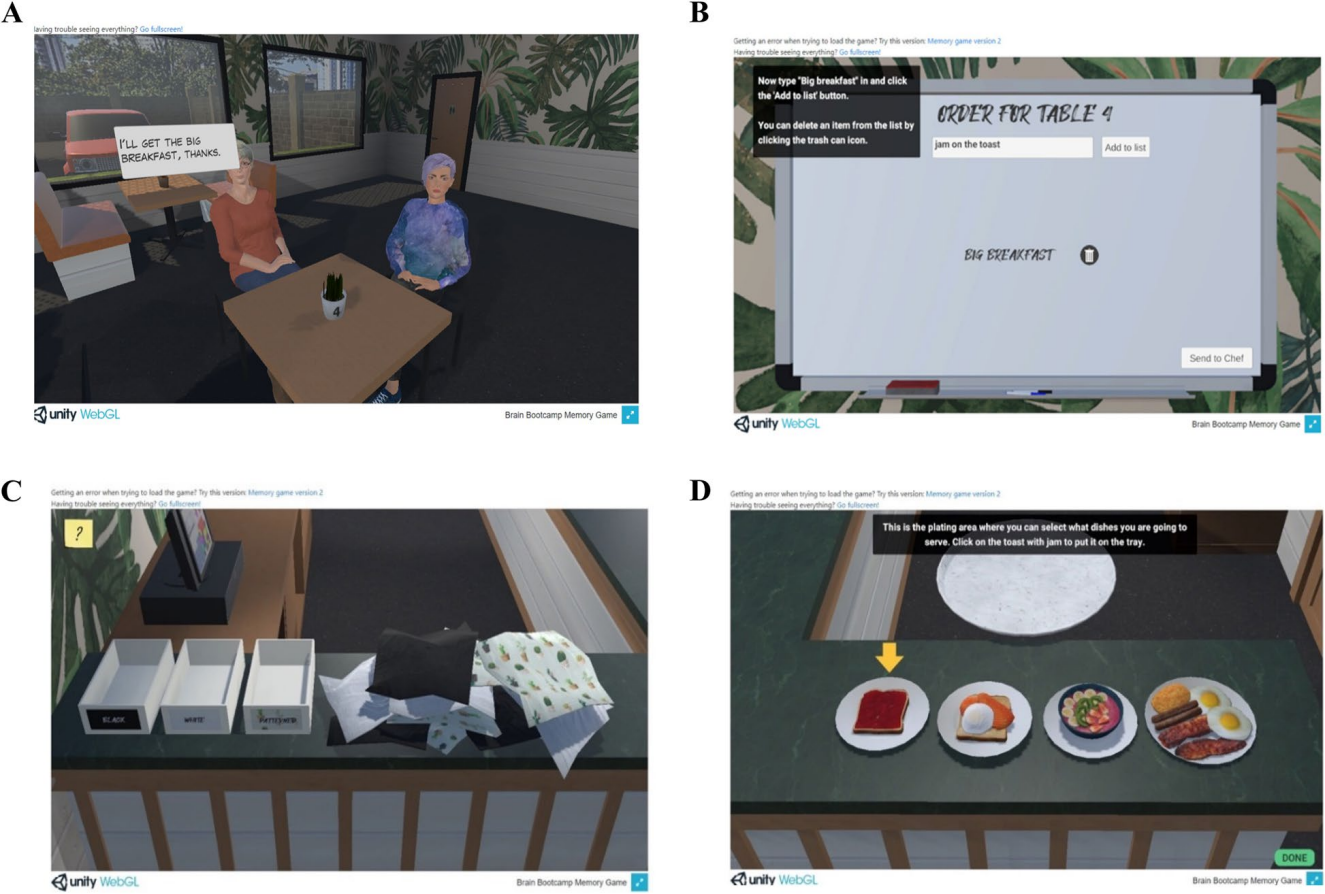
Graphical details of various scenarios in the Leaf Café module. **A** Initial presentation of two customers at a table. **B** Whiteboard for participant to record customer order. **C** Example of a distraction sorting task. **D** Food counter for participants to select food items matching order given in A

The module was designed to simulate a real environment that allows participants to deliver food or drinks at the café, activities that serve to assess various aspects of the participants’ cognitive function including processing speed, learning and memory, and executive functioning. The user was required to complete a series of actions that emulate a range of service activities. We presented the user with four tables with two people at each table (Fig. 1A). The user was asked to remember the table and the customers’ order/s and subsequently report them to the chef (Fig. 1B). Following this, a distraction task (e.g., napkin sorting) of approximately 120s was presented to the participant (Fig. 1C) before the chef asked the participant to re-record the customers’ order/s due to an unforeseen error in the kitchen. Finally, participants were asked to select the correct dishes from the counter (Fig. 1D) and serve them to the correct customers. The module was designed to have five levels, whereby each increase in level equates to a higher memory load of customer order (i.e., 2, 4, 6, 8, and 16 orders for each respective level). Participants firstly completed a short tutorial and were informed of the requirements of each action to familiarise users with the mechanics of the system.

At the end of the module, participants obtained a score out of 180, which was achieved by the participant serving up to four tables of customers correctly (see detailed scoring algorithm in Table 1) and reflected the participant’s performance on each Leaf Café component. Four relevant tasks were selected and scored depending on the participants’ ability to complete the task correctly, the number of attempts, and the proportion of tasks performed correctly. Although arguably tapping into perceptual-motor and executive skills, performance was ultimately assessed in terms of memory formation and retention, scored manually using the scoring algorithm (Table 1) in the following order:

1. Immediate recall of customer’s order (immediate recall)
2. Delayed recall of customer’s order (introduced in Level 1 onwards; delayed recall)
3. Selection of correct meals from a range of options presented on a counter (delayed recognition)
4. Serving the dish to the correct table and customer (spatial memory)

**Table 1:** Scoring algorithm of the LEAF CAFÉ module

**Level**	**Immediate Recall Score**				**Delayed Recall Score**				**Delayed Recognition Score**				**Spatial Score**			**Customer Recall Score**			
	No Attempt	Per item	Per drinks	Total points available	No Attempt	Per item	Per drinks	Total points available	No Attempt	Per item	Per drinks	Total points available	No Attempt	Per item	Total points available	No Attempt	Per item	Total points available	Total points available per level
**1** 2 food items	0	1	0	2	0	1	0	2	0	1	0	2	0	1	2	0	1	2	10
**2** 4 food items	0	1	0	4	0	1	0	4	0	1	0	4	0	1	4	0	1	4	20
**3** 6 food items	0	1	0	6	0	1	0	6	0	1	0	6	0	1	6	0	1	6	30
**4** 8 food items	0	1	0	8	0	1	0	8	0	1	0	8	0	1	8	0	1	8	40
**5** 8 food + 8 drink items	0	1	1	16	0	1	1	16	0	1	1	16	0	1	16	0	1	16	80
**Total points available**				36				36				36			36			36	180

If an individual exhibited poor performance on any level (as defined by < 50% accuracy for all four metrics listed above), discontinuation was initiated and participants were guided to the final section of the module (i.e., “Thank you for helping us out today”). Thus extended gameplay typically indicated advancement to the next level and the chance to achieve a higher score. In the module, accuracy across the above domains was the main outcome measure. The focus was on ensuring precise and correct processing of information rather than emphasising speed.

Participants completed a 19-statement online questionnaire (Supplementary File) to assess their experience and overall perception of the game.

Demographic questions were presented first including gender, age, employment status, marital status, living status, education, country of birth, background, and income. Then, a total of 11 questions were asked to gain feedback regarding the ease of use of the module. These questions consisted of both close-ended and open-ended questions. Close-ended questions asked participants to respond on a 5-point Likert scale on the enjoyability and feasibility of the module (strongly agree to strongly disagree), communication and instructions of the module (very good to very poor), and the degree to which participants felt supported during the module (very supported to very unsupported). Open-ended questions targeted the usefulness of the module, suggestions for changes, and reasons for non-recommendations. Dichotomous questions (e.g., Yes, No) were also present to gauge the recognisability of the foods (*n* = 31) and drinks (*n* = 9) within the module. Finally, a forced choice question was presented at the end regarding whether or not the participant would recommend the module to others. The feedback survey took approximately 10 – 15 min to complete.

### Statistical analysis

Sociodemographic characteristics were compared between groups using a two-sample t-test and the Fisher exact test for continuous and categorical variables, respectively. Summary statistics were calculated individually for the module performance and survey responses.

Module performance scores were analysed at each task level, given a total score, and compared between the two groups using the methods described above, as appropriate. The module captured multiple variables including time, number of correctly and incorrectly recalled items at immediate and delayed recall, number of correct and incorrect items selected at the counter, number of correctly and incorrectly identified tables and customers, and total number of correctly performed tasks. The start and end time of each task was recorded and was used to calculate the participant’s time on each individual component of the module, which also included the time spent on the practice items.

Relationships between traditional memory performance scores (TICS-M) and other module variables (e.g., total score, immediate and/or delayed recall) were assessed using Pearson correlations, and a *p*-value of < .05 was considered statistically significant. Analyses were performed using SPSS (Version 25).

The content analysis, employing iterative and inductive techniques [39], focused on evaluating participants' responses related to cognition, acceptability, likability, and suggested improvements within our cognitive assessment module. Led by JS with continuous input from co-authors, the process involved exporting survey comments (*n* = 57) to an Excel file. Firstly irrelevant comments were excluded, covering issues such as module comprehensiveness and miscellaneous suggestions. Open coding was then applied, condensing meaning units, grouping similar content under higher-order headings to develop codes, and importing them into NVivo 17 for creating higher-order categories. In the final abstraction phase, descriptive summaries and responses were assigned to each category, forming subcategories when applicable. A codebook was also generated to index text and meaning between two raters (JS and JG). All data underwent independent coding, and inter-rater agreement was assessed using the Kappa coefficient (k), categorised by parameters: 0.8–1.0 = almost perfect; 0.6–0.8 = substantial; 0.4–0.6 = moderate; 0.2–0.4 = fair [40].

## Results

### General characteristics

Sample characteristics are shown in Table 2. The age of the sample (*N* = 131) ranged from 20 to 85 years, with an average age of 54.9 years (SD = 20.8). Two subsamples were derived based on age with younger adults identified as participants < 65 years old (*N* = 56, M = 33.7, SD = 12.8) and older adults as ≥ 65 years old (*N* = 65, M = 71.2, SD = 4.5). Around three-quarters of participants were female (71.8%) and lived in a metropolitan area (83.8%). Most participants had high levels of education, with 79.8% reporting educational attainment of more than 12 years. Participants were of high socioeconomic status, with around three-quarters scoring in the highest three quintiles (78.5%). There were significant differences between the subsamples in terms of country of birth, with more younger adults born from a non-English-speaking country compared to older adults (50.9% vs 2.8%; *p* < .001).

**Table 2:** Demographic summary of sample

Variables	All N (%)	Younger Adults N (%)	Older Adults N (%)
Gender*			
Female	94 (71.8)	34 (61.8)	60 (80.0)
Male	37 (28.2)	22 (38.2)	15 (20.0)
**Age (Mean [SD], Range)**	54.9 [20.8], 20–85	33.7 [12.8], 20–63	71.2 [4.5], 65–85
18–24	20 (15.27)		
25–34	14 (10.69)		
35–44	10 (7.63)		
45–54	8 (6.11)		
55–64	5 (3.82)		
65–74	56 (42.75)		
75–84	17 (12.98)		
85–94	1 (0.76)		
Education*			
Low	6 (4.8)	0 (0.0)	6 (9.0)
Middle	19 (15.3)	6 (10.5)	13 (19.4)
High	99 (79.8)	51 (89.5)	48 (71.6)
**Locality**			
Metropolitan	109 (83.8)	51 (91.1)	58 (78.4)
Regional/Remote	21 (16.2)	5 (8.9)	16 (21.6)
**SES Quintiles**			
1 (lowest)	12 (9.2)	6 (10.7)	6 (8.1)
2	16 (12.3)	5 (8.9)	11 (14.9)
3	20 (15.4)	4 (7.1)	16 (21.6)
4	22 (16.9)	14 (25)	8 (10.8)
5 (highest)	60 (46.2)	27 (48.2)	33 (44.6)
Country of birth***			
English Speaking	97 (75.8)	28 (49.1)	69 (97.2)
Non-English Speaking	31 (23.2)	29 (50.9)	2 (2.8)
**Cognitive Score (mean, [SD], range)**	27.1, [3.7], 20–39	26.7, [3.1], 20–36	27.4, [4.1], 20–39
Impaired	5 (4.0)	2 (3.9)	3 (4.1)
Not impaired	120 (96.0)	49 (96.1)	71 (95.9)

Denotes significant difference in proportion between younger adults and older adults

^*^p < .05

^**^p < .01

^***^p < .001

### Acceptability and feasibility

**Fig. 2:**
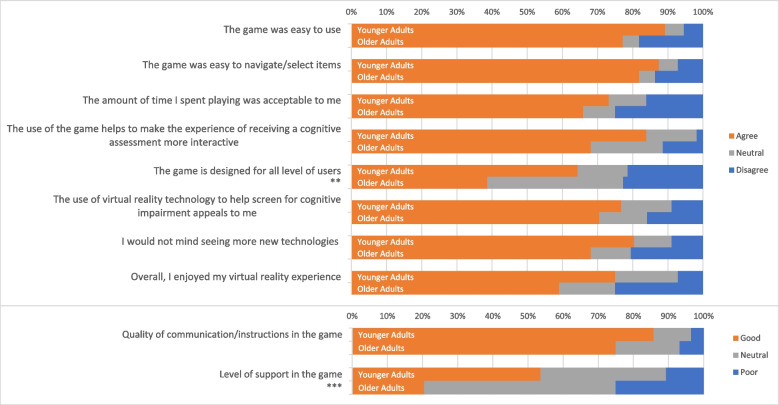
Proportion of responses for younger adults and older adults on user experience. **p <.01, ***p <.001

Overall, most participants (*N* = 100/130; 76.9%) provided a response on their their user experience (Fig. 2). Most participants agreed that the module was easy to use (84%), easy to navigate and select items from (85%), and found time spent playing was acceptable (70%). About two-thirds of participants reported enjoying their virtual reality experience (68%).

Approximately three-quarters of participants agreed that the use of virtual reality technology made screening for cognitive impairment more appealing (74%), the use of the game made cognitive assessment more interactive (77%), and that they would not mind seeing more new technologies (75%). Additionally, 81% of participants were satisfied with the quality of communications and instructions in the game.

There were significant differences between the proportion of responses between younger adults and older adults on two user experience items. A higher proportion of younger adults agreed that the game was designed for all users (64.3% vs 36.8%; *p* = .020). Additionally, a larger proportion of younger adults reported feeling supported in the game when compared with older adults (53.6% vs 20.5%; *p* = .007).

### Recognition of items

**Fig. 3:**
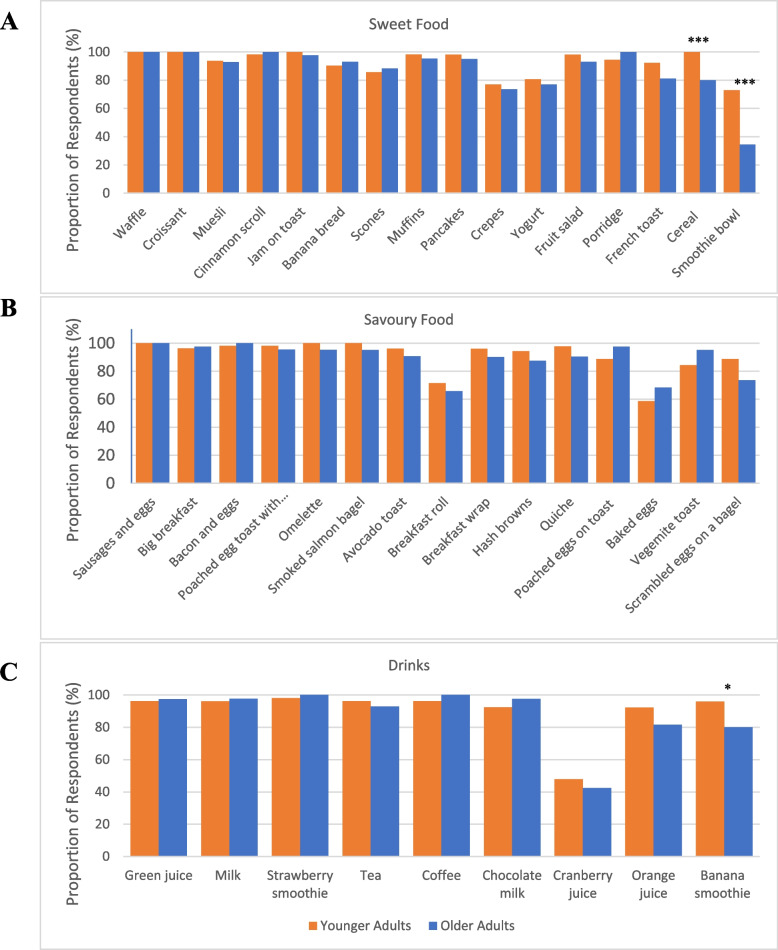
Percentage of recognition of module menu items sorted by recognition difference between younger adults and older adults (smallest to largest). *p <.05, **p <.01, ***p < .001

The proportions of respondents who recognised the food and beverage items are presented in Fig. 3. There were three classifications, which included savoury food items, sweet food items, and beverages. On average, most food and beverage items were well recognised by both age groups, with an average recognition of 89.8% for all items. The highest recognition items across both age groups were sausages and eggs, waffle, and croissant (100%), and the lowest recognition item was cranberry juice (45.1%). There was a significant age difference across recognition, where younger adults had higher levels of recognition of the smoothie bowl (72.9% vs 34.4%), cereal (100% vs 80%), and banana smoothie (95.9% vs 80%) than older adults (*p* < 0.05). However, filler items, which were not presented for orders were also included in the module and had low levels of reported recognition. These food and beverage items were only introduced at the counter and included the smoothie bowl, smoked salmon and poached egg on toast, and cranberry juice.

### Cognitive performance: TICS-M

The average TICS-M score for all participants was 27.1 (SD = 3.7, range 20–39). A total of five participants (all older adults) scored below the threshold for possible cognitive impairment (≤ 21), however, were still included in the study. There were no significant differences in mean total TICS-M scores between younger (M = 26.7, SD = 3.1) and older (M = 27.4, SD = 4.1) adults (*p* = .34).

### Virtual reality module: Leaf Café

Across the sample, participants spent an average of 32.8 min playing the game (SD = 13.3) with the vast majority of participants completing four levels or more (92.2%). No significant difference between time spent on the module was found with older adults spending an average of 34.5 min compared to the younger adult average of 30.3 min (*p* > .05).

#### Total score

The highest total potential score for participants was 180. The mean module score was 107.6 (SD = 38.7, range 2–180), and younger adults produced higher scores compared to older adults (M = 124.1 vs 95.9; *p* < .001).

#### Total sub-category scores

Younger adults also had significantly higher scores compared to older adults on all four subdomains including immediate recall, delayed recall, delayed recognition, and spatial score (all *p*s < .001) (Table 3).

**Table 3:** Summary of the LEAF CAFÉ module functionality and scores

VR Component	All N (%)	Younger Adults N (%)	Older Adults N (%)
Mean total time playing the game (minutes [SD], range)**	32.8 [13.3], 1.52 – 75.27	30.3 [9.2], 18.73–68.46	34.5 [15.3], 1.52–75.27
Levels Played (mean levels [SD], range)***	4.66 [.81], 1–5	4.98 [0.14], 4–5	4.43 [0.99], 1–5
1	1 (0.8)	0 (0.0)	1 (1.4)
2	6 (4.7)	0 (0.0)	6 (8.1)
3	3 (2.3)	0 (0.0)	3 (4.1)
4	15 (11.7)	1 (1.9)	14 (18.9)
5	103 (80.5)	53 (98.1)	50 (67.6)
VR Total Score (mean [SD])***	107.6 [38.7]	124.4 [26.3]	95.9 [41.6]
VR Total Immediate Recall Score (mean [SD])***	24.9 [9.3]	29.4 [4.0]	21.8 [10.6]
VR Total Delayed Recall Score (mean [SD])***	18 [8.4]	21.5 [6.6]	15.6 [8.7]
VR Total Delayed Recognition Score (mean [SD])***	29.5 [8.7]	33.1 [3.3]	27 [10.2]
VR Total Spatial Score (mean [SD])***	36.8 [17.2]	44.7 [15.3]	31.3 [16.3]

Denotes significant difference between younger adults and older adults

^*^p < .05

^**^p < .01

^***^p < .001

#### Sub-category scores at each level 1–5

**Fig. 4:**
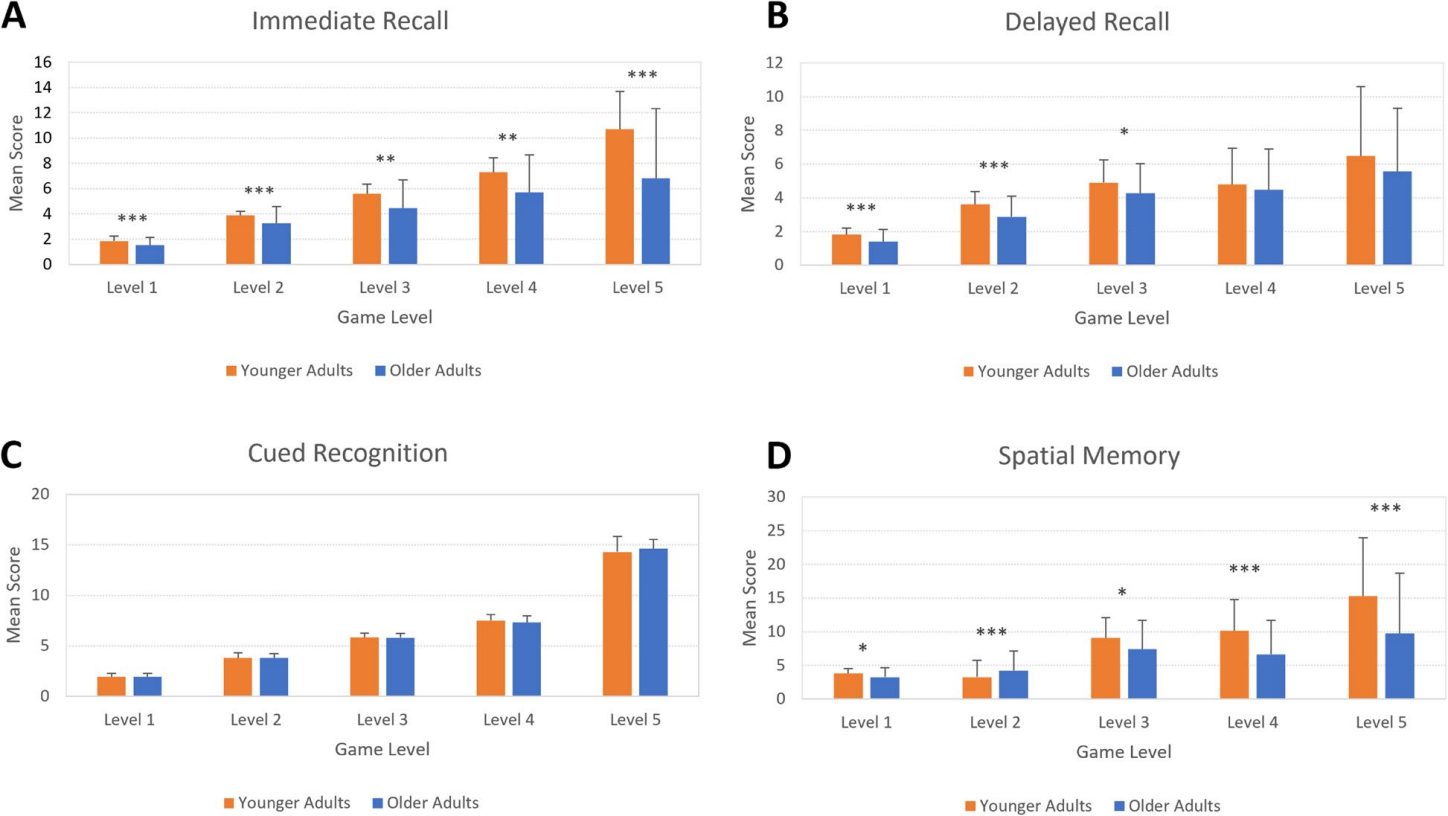
Summary of VR scores between younger adults and older adults on sub-scale categories

Younger adults produced significantly higher scores from Level 1 to 4 (*p* < .05). They also had significantly higher scores in most subdomains, including immediate recall, delayed recall, and spatial memory across various game levels (*p* < .05). There were no significant age differences between scores for cued delayed recognition (*p* > .05) (Fig. 4).

### Correlation between TICS-M and Leaf Café module

**Fig. 5:**
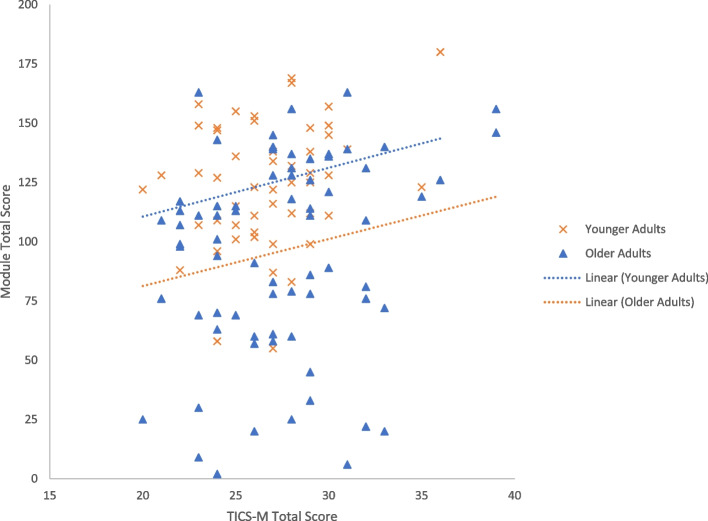
Correlation of TICS-M and module total scores for younger adults and older adults

Across the sample, total scores of the TICS-M and the module were positively and significantly correlated (*r(123)* = *0.16*, *p* = .038) (Fig. 5). There were also significant positive correlations between the TICS-M total score and Leaf Café total score specific to younger (*r*(49) = 0.24, *p* = .042), and older adults (*r*(72) = 0.19, *p* = .049).

#### Qualitative feedback

Three main themes emerged regarding participant experiences, which focused on positive experiences and enjoyment, difficulties experienced, and future directions.

##### Theme 1: Positive experiences and enjoyment

Many participants expressed positive feedback and satisfaction with the study, highlighting a sense of engagement and appreciation for their contribution to the university. As highlighted by participants, *"No, you are doing a great job" *and* "I am delighted to be part of this experiment."* Furthermore, participants acknowledged the game as an excellent memory test, supporting participants’ self-awareness regarding their cognitive strengths and areas that require improvement. As mentioned by a participant*, "Great memory test! Reveals my need to focus."* Participants also highlighted the module’s effectiveness in stimulating thinking and stretching the mind, providing a cognitive challenge and engagement of higher-order cognitive processes. Exemplifying this theme, a participant expressed, *"It made me think, I am sure I didn't do as well as I thought."*

In general, whilst participants enjoyed the module there were diverse opinions on enjoyment and perceived usefulness, and these discussions demonstrate the varied impacts of the game module on participants’ reflections regarding memory capabilities, learning preferences, and challenges associated with memory retention.

Some participants found value in taking breaks from the game, going for a walk, and returning refreshed. This approach was perceived as aiding in memory recall. As noted by a participant, *"I left the game and went for a walk and came back refreshed and maybe remembered most of it [the orders]."*

The clarity and engagement provided by the game's instructions were also identified as valuable. Participants appreciated instructions that maintained interest throughout all levels, *"Instructions, it kept my interest through all the levels."*

##### Theme 2: Difficulties experienced

Participants commonly mentioned the difficulty in remembering multiple tables and associated drink orders, expressing challenges and strain on their memory. One participant noted, *"I found it very hard with trying to remember all 4 tables and what drinks went with what,"* reflecting the cognitive demand experienced. Challenges in memory were also expressed, with a participant noting feeling uncomfortable due to difficulties remembering orders. As a participant described, *"Felt very uncomfortable because it was evident that I couldn't remember the orders."*

These challenges were often associated with the lack of clear instructions and feedback mechanisms within the game. Participants felt uncertain about what to do if they couldn't remember parts of an order, leading to frustration. One participant expressed, *"Lack of instruction for what to do if you didn't remember some or all of an order,"* highlighting the need for more explicit guidance as well as expectation setting (e.g., it is not expected or typical for people to be able to remember all the orders). Participants further expressed uncertainties and a lack of clarity about their performance, particularly regarding correctness in their memory choices. A participant described, "*Not knowing if I was correct in my memories, so I could work with elimination as a method," *indicating a need for clearer feedback.

A recurring subtheme revolved around technical issues and challenges related to the user interface. Participants mentioned concerns about screen size, navigation problems, and difficulties accessing the game on certain devices. One participant noted, *"Not sure if it was my computer or the game, but was put off initially by the graphic being too big for my screen."* Participants also expressed difficulties with the game’s initial confusion due to a large screen and the need to scroll for instructions. A participant reported, *“A little confusing at first. The screen was too big, so had to zoom down to be able to read instructions on both sides.”*

Similar issues related to visual recognition and instructional challenges were also raised by participants. Some found it difficult to differentiate food items, while others experienced challenges with the game's instructions. A participant shared, *"Connecting what each item looked like with the memory of what had been ordered wasn't always easy because you couldn't picture the dish when the person ordered."* Furthermore, issues related to clarity in instructions and feedback mechanisms within the game were also expressed. Participants conveyed a need for clearer instructions, particularly in scenarios where they could not remember parts of an order. One participant recommended,* "Add (delayed) instructions for what to do if you can't remember what to write."*

Participants generally expressed varied levels of awareness regarding the music. Some acknowledged the presence of music without being distracted, while others found it relaxing and contributing positively to the game's realism. The concept of background noise and a realistic atmosphere was generally well-received. One participant noted, *"They added a quality of realness to the game that was great. It helped to make the effect positive and useful."* Some participants appreciated the realistic background noise, finding it inoffensive and contributing to the lifelike experience of being in a cafe. Another participant mentioned, *"Appropriate sound, make it more lifelike and realistic."* However, the impact of music on the gaming experience was mixed, with some perceiving it as a positive addition and others finding it distracting. For instance, there were instances where participants felt that the realism became a distraction, impacting their engagement with the game. A participant stated, *"Rather annoying. I turned the sound off for the last exercise as I found it distracting."*

Finally, some participants mentioned aspects related to the duration and repetitiveness of the game. Some found the game too lengthy or monotonous, impacting their engagement. Concerns about the duration of the game and the pacing of activities were mentioned. Some participants felt that the game was too long in one sitting, leading to boredom and annoyance. Suggestions included reducing the number of ordering sessions and incorporating breaks during the game. A participant commented, *"It's too long in one sitting. I got bored and then annoyed because I had 'better' things to do like deal with real-world issues."*

##### Theme 3: Future directions

Participants provided helpful insights into their experiences with the game module and offered constructive recommendations for future improvements. Subthemes centred around technical considerations, cognitive demands, and a desire for varied and realistic gaming scenarios.

Participants expressed interest in exploring varied game dynamics and realistic scenarios. Suggestions included a more gradual lead-up in difficulty levels, more recognizable meals and drinks, and the option for assistance within the game. A participant observed, *"Perhaps provide the option of assistance, so that the waiter could ask to see the menu to remind herself of the items available."*

Several participants mentioned the cognitive demand and strain on memory, suggesting the need for adjustments in game dynamics. Some expressed the desire for more time to read instructions or felt that the game escalated too quickly, leading to frustration and boredom. A participant commented, *"The range of dishes gave clues, but generally the game escalated too quickly to four tables and mixed orders."*

Several participants highlighted the importance of inclusive design, considering the needs of different user groups. Recommendations included accommodating various screen sizes, providing options for auditory cues, and ensuring that instructions are clear and accessible to all users, regardless of their technological proficiency. A participant suggested, *"Make your program sense the size of the user's screen so that your pictures fit on it.”*

## Discussion

Leaf Café provides a feasible and acceptable method for assessing cognitive function, offering an engaging and ecologically relevant approach. Participants reported positive experiences with the module, finding it acceptable, easy to use and navigate. Crucially, the scores from the module demonstrated small but significant correlations with scores obtained from a traditional cognitive screening tool, indicating its potential utility in detecting cognitive impairment.

Our study capitalises on the ability to offer an interactive immersive environment to participants that can be used on their own personal computers. In contrast, past studies exploring older adults’ acceptability of virtual reality was limited to fully-immersive VR platforms [41, 42] that require specialised hardware and technical support. By designing a module that is supported by most web browsers, we have been able to recruit a larger sample for the current study. This is in contrast to the smaller sample sizes (*N* < 30) evident in existing studies on VR assessment, which make it hard to generalise conclusions from these studies more broadly [23–25]. Furthermore, studies based on novel immersive VR platforms offer unfamiliar and heavy setups that may cause discomfort and motion sickness [43]. Comparatively, this study was able to capture the positive experiences of both younger adults and older adults to validate the feasibility of Leaf Café module for cognitive screening. Additionally, our study revealed possible age-related differences in recognition among the menu items. Whilst there was a generally high level of recognition, certain food and beverage items exhibited variability in recognition rates across different age groups. Perhaps incorporating a pre-selection section, where participants engage in a recognition task for various items, could inform the selection of items used in the game. This approach would not only enhance the relevance and effectiveness of the module but also allow for the consideration of cultural differences in exposure to different food items. These adjustments would contribute to refining the module and addressing potential age-related differences in recognition rates..

Existing pen-and-paper-based cognitive screeners often deviate from real-life scenarios, reducing the ecological validity, thus hampering their ability to predict functional impairment [44]. The Leaf Café module is modelled on a natural setting, providing increased ecological validity, which improves the generalisation of findings to real life [45, 46]. Additionally, the module enables the collection of a wider variety of data, collected in real-time, which can increase the sensitivity of the screener to discriminate differences in participant performance when compared to conventional cognitive screening tools [32, 47]. Being software-only and not hardware dependent, our tool is relatively easy to customise for specific populations even within the same module and session or addition of screeners and tasks. Our results suggest that participants enjoyed the virtual reality environment and saw its potential as an early cognitive assessment tool. These findings provide a foundation for future research using different computerised environments to assess cognition, though accurate and sensitive detection of changes in cognitive status remains an area for further study in the field of VR.

The discrepancy in performance between older and younger adults within the module echoes a trend often observed in traditional cognitive assessments. Specifically, the lower advancement rates of older adults in reaching higher levels within the module align with their typically lower performance on established cognitive tests [48–51]. While it is important to note that this correlation does not imply a direct causation between the two, it does suggest a potential parallel in cognitive functioning across different assessment modalities. However, it is essential to acknowledge that this observed pattern does not definitively establish a causal relationship between performance in the module and outcomes on traditional cognitive assessments. Future studies could explore deeper the underlying factors contributing to age-related differences in performance across various cognitive tasks. Additionally, investigating how other demographic and individual characteristics may influence performance in both the module and traditional cognitive assessments would provide a more comprehensive understanding of cognitive functioning across different age groups. Such research efforts could offer valuable insights into the utility and validity of the module as a cognitive assessment tool and guide further efforts to enhance its effectiveness in evaluating cognitive abilities across the population.

Indeed, the substantial range of scores observed in our module, spanning from 2 to 180, invites an exploration of the factors contributing to such variability. One plausible explanation could be the influence of several participants who screened below the threshold for possible cognitive impairment. Undeniably, cognitive assessments, by their nature, are susceptible to individual differences, and various factors, including age, education, and cognitive reserve, can contribute to the observed diversity in scores [52]. The wide range identified in our study prompts reflection on the sensitivity and specificity of the module assessment tool employed and to consider balancing sensitivity to detect cognitive decline and specificity to avoid misclassifying individuals as well as supervising sessions to ensure proper engagement in the task.

Whilst our survey had a 75% response rate, the decision not to participate in the survey may itself signify a form of feedback, indicating varying degrees of engagement or interest in providing evaluative input. While the specific reasons for non-participation remain unknown, this lack of response prompts consideration of potential motivations behind participants’ choices, as individuals who voluntarily engage in post-intervention evaluations may possess a predisposition towards positive appraisals [53]. Therefore, the non-response rate could serve as another indicator of participants' level of endorsement or reservation towards the game module. Further exploration of this aspect could enhance our understanding of participant engagement and its implications for the interpretation of feasibility and acceptability outcomes.

The observed divergence in perceptions between younger and older adults regarding the game's design and support introduces considerations when designing such tools. It is conceivable that younger adults, potentially more adept at navigating digital interfaces and gaming environments [54–56], may find the design intuitively accommodating. Conversely, older adults, who may possess less familiarity with such interfaces, could perceive certain elements as less tailored to their preferences [56]. Conducting targeted user feedback sessions or making iterative design adjustments based on age-specific preferences may serve to address this divergence. Additionally, exploring the subtle influences of self-perceived cognitive abilities, even within a cognitively unimpaired sample, could provide additional information on participants' attitudes towards the cognitive testing aspect of the game. Further investigations into these topics hold promise for uncovering insights that facilitate a more inclusive and universally user-friendly design for individuals of all age groups.

Little is known about the ability of VR technology to successfully discriminate between cognitively impaired and non-impaired groups. Our study did not attempt to use VR cognitive scores to discriminate between such groups, with only a few participants producing TICS-M scores placed below the threshold defining possible cognitive impairment. However, scores on the TICS-M were correlated with module scores for both age groups, suggesting its potential as a cognitive screening tool. Correlations were small, possibly due to different scopes in focus across the tests: the TICS-M included items which were likely to be scored with 100% accuracy in a putatively healthy sample (e.g., in orientation and language domains), while the module scores reflected memory formation and retention across a variety of difficulty levels.

### Implications

VR technology may ultimately provide clinicians with the ability to identify and track progression of cognitive decline for their patients, and by doing so, will make such assessments more accessible and cost-effective and providing greater clarity regarding prognosis and intervention planning. Our study provided novel insights into the feasibility and acceptability of virtual reality platforms for measuring cognition in younger adults and older adults that could be implemented in health and clinical settings (e.g., primary care, aged care) to monitor and track the cognitive health of patients and residents. Such tools may also be useful as preliminary screeners to provide respondents with initial insights into possible cognitive concerns requiring clinical follow-up. Improving the accessibility and acceptability of cognitive screening tools using VR platforms increases the likelihood of early detection of cognitive impairment, allowing clinicians improved opportunities for early intervention. Our study was able to heighten the appreciation of the use of VR in carrying out cognitive tests. Moreover, the construction and calibration of our non-immersive VR module was user-friendly and made many of our participants believe that there is a future for this technology within the clinical setting.

### Limitations

Being a feasibility study primarily aiming to evaluate the acceptability of virtual reality as a screening tool, we did not seek to enrol participants with cognitive impairment (indeed, diagnosed dementia was an exclusion criterion), and future studies should include participants with clinically validated impairment and/or diagnoses. Our module was also only validated against a single diagnostic cognitive screener, which limits comparisons, restricting the scope of our assessments to the parameters and criteria to the TICS-M only. This narrow focus may limit the generalisability of our findings and their applicability to a broader range of cognitive assessment tools or contexts. Future studies would likely benefit from validation against additional cognitive screeners and other gold standard cognitive assessment tools. Furthermore, given the diversity in the broader population, future studies should seek to establish the validity of the assessment in participants from socioeconomic, education, cultural, and linguistically diverse backgrounds.

Another limitation of this study is the remote nature of the module administration, which introduces uncertainty regarding the potential assistance participants may have received in completing the task. The lack of direct supervision raises questions about the independent execution of the cognitive screening, which could impact the reliability of the results. Future research endeavours could investigate the implementation of this tool through group administration under supervision, which holds the potential to be an efficient model compared to current methods.

Finally, the variability in screen sizes among participants' own devices posed a significant challenge in design, as catering to small screens alone would likely compromise the immersive experience. Immersion levels are closely linked to screen size, making it essential to address this variability effectively. While a Head-Mounted Display would provide greater immersion by encompassing the user's entire field of view, the research team chose to use a screen for practical reasons and to mitigate concerns regarding motion sickness. Future studies should explore strategies to optimise immersion levels across different screen sizes, while also investigating alternative immersive technologies that could enhance user experience without sacrificing practicality or inducing adverse effects like motion sickness.

## Conclusion

In conclusion, we demonstrated that Leaf Café is a feasible and acceptable tool for cognitive assessment, offering an engaging and ecologically relevant approach to evaluate cognitive performance. However, research is needed to further validate the utility of the game in detecting clinically significant cognitive impairment. Future studies should focus on establishing the diagnostic accuracy and sensitivity of the module for different clinical populations and comparing its performance to traditional cognitive assessment tools, thereby contributing to the growing evidence base for virtual reality-based cognitive assessments.

### Supplementary Information

**Supplementary Material 1.**

## Data Availability

Composite data can be made available upon reasonable request to the corresponding author (joyce.siette@westernsydney.edu.au).
