# Supplementary material for: Development of a new computer simulated environment to screen cognition: assessing the feasibility and acceptability of Leaf Café in younger and older adults
Source: BMC Med Inform Decis Mak. 2024 Mar 19;24:79. doi: 10.1186/s12911-024-02478-3 (PMC10949698; doi:10.1186/s12911-024-02478-3)
Supplement: Supplementary file 1 — Supplementary Material 1. [file 12911_2024_2478_MOESM1_ESM.docx]

**Supplementary File**

**Feedback Questionnaire**

For the following questions, please select how much you agree with the statement about the game.

- 'The game was easy to navigate/select items.'

- 'The game was easy to use.'

- 'The amount of time I spent playing the game was acceptable to me.'

'The use of the game helps to make the experience of receiving a cognitive assessment more interactive.'

- 'The game is designed for all levels of users.'

- 'The use of virtual reality technology to help screen for cognitive impairment appeals to me.'

- 'I would not mind seeing more new technologies like this game being used by health care professionals (e.g., doctors) during a consultation.'

- 'Overall I enjoyed my virtual reality experience.'

Can you let us know approximately how long it took for the game to load?

How did you feel about the time you spent waiting to access the online game?

How did you find the quality of communication/instructions in the game?

How supported did you feel whilst in the game?

Did you play the game with sound?

What were your thoughts about our sound effects? These could be positive or negative.

What did you find most useful about the game?

What did you find least useful about the game?

Can you suggest any changes?

We would like to know if the food on the menu is widely recognised. We have an image of each food order with its corresponding label. Please select 'yes' if you think the food matches its label accurately.

We now have some images of each drink order with its corresponding label. Please select 'yes' if you think the drink matches its label accurately.

And our final questions.

- Would you recommend this memory game to your friends or family members?
- Why would you not recommend this memory game to your friends and family members?
